# Supplementary material for: Inhibition of Histone Deacetylase Activity Increases Cisplatin Efficacy to Eliminate Metastatic Cells in Pediatric Liver Cancers
Source: Cancers (Basel). 2024 Jun 22;16(13):2300. doi: 10.3390/cancers16132300 (PMC11240720; doi:10.3390/cancers16132300)
Supplement: Supplementary file 1 [file cancers-16-02300-s001.zip › cancers-3033186-supplementary.pdf]

Gulati et al:

Supplemental Figure S1 and Whole Images of Gels

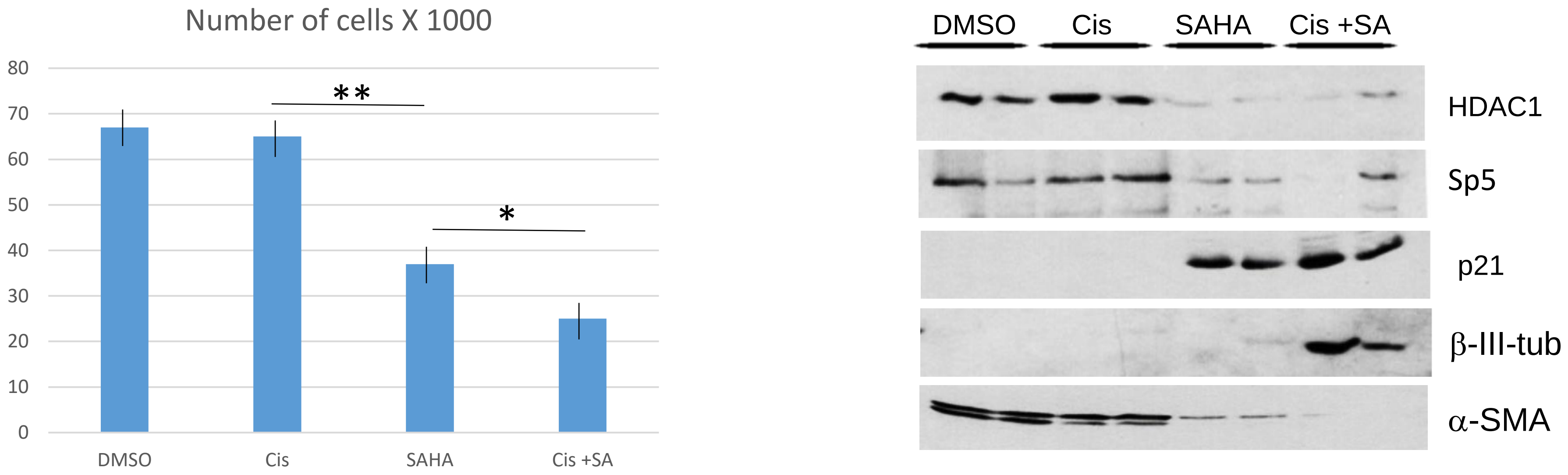

Gulati: Supplemental Figure S1. Treatments of hbl107 line with DMSO, Cisplatin , SAHA and combination of cisplatin + SAHA. Left part: Bar graphs show number of cells at 48 hours after initiation of treatments. The data are collected from triplicate plates. *$p < 0.05$, **$p < 0.01$. Right part: Western Blot shows levels HDAC1, Sp5, p21, β-III-tubulin and α–SMA in cells treated with the drugs.

File S1. WHOLE GEL IMAGES

Fig 1D

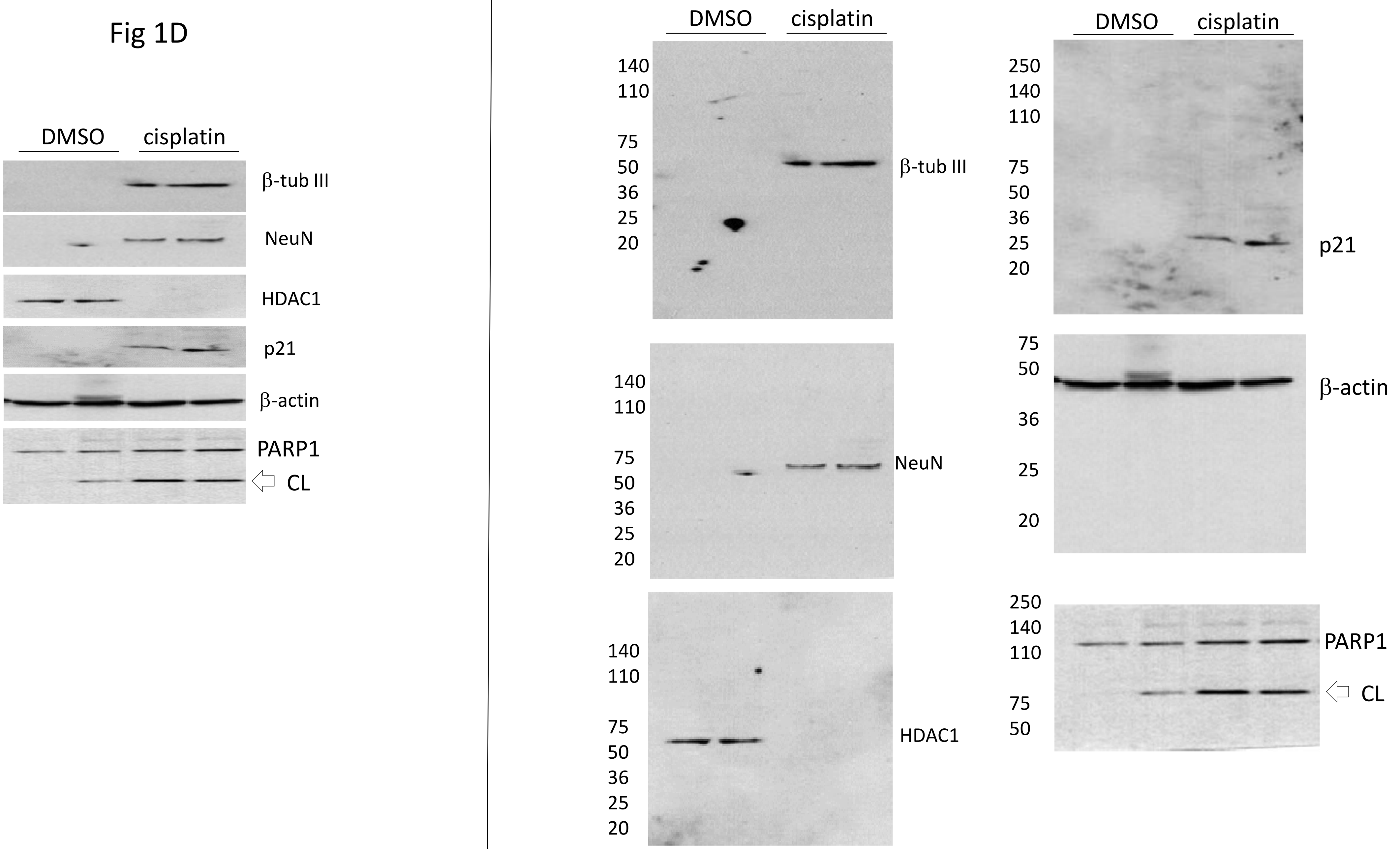

Whole Gel Images

Fig 4A

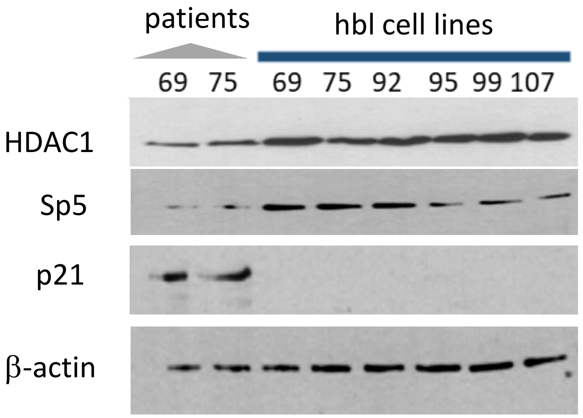

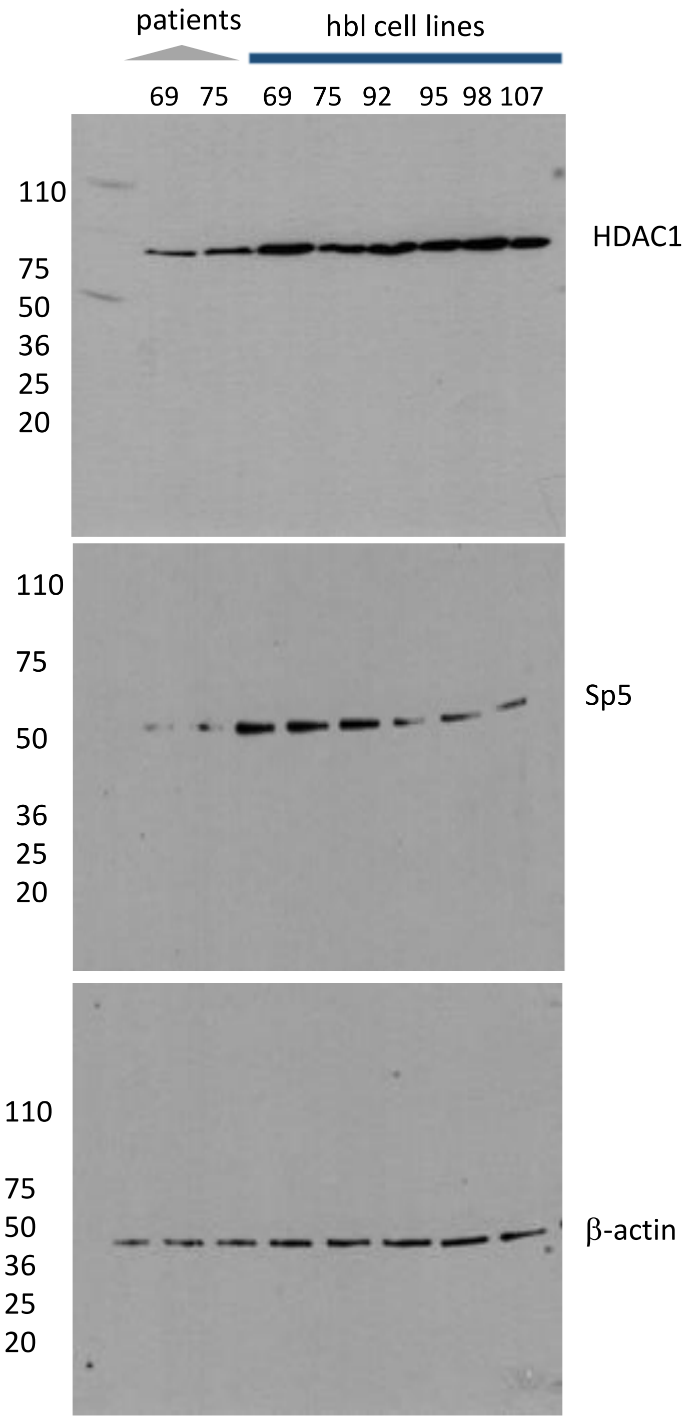

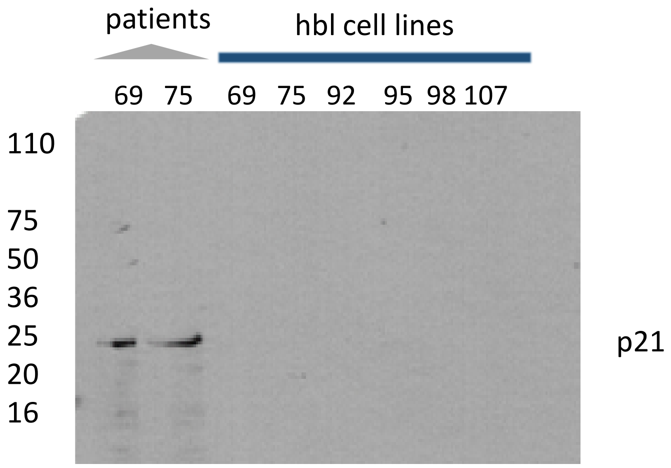

Whole Gel Images

Fig 4E

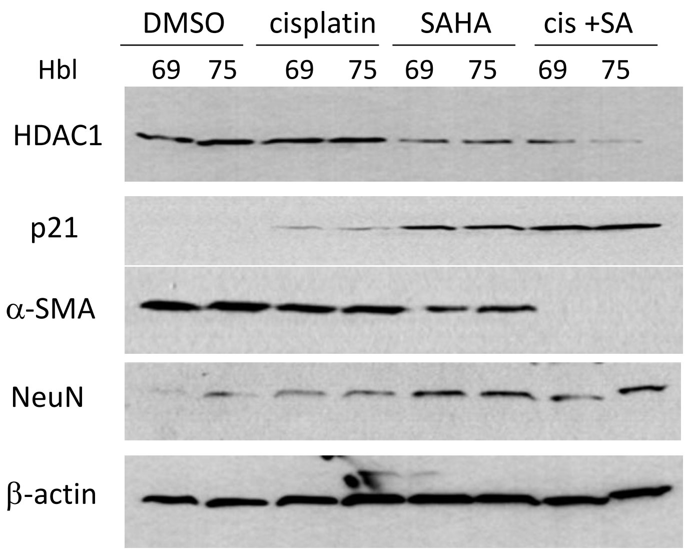

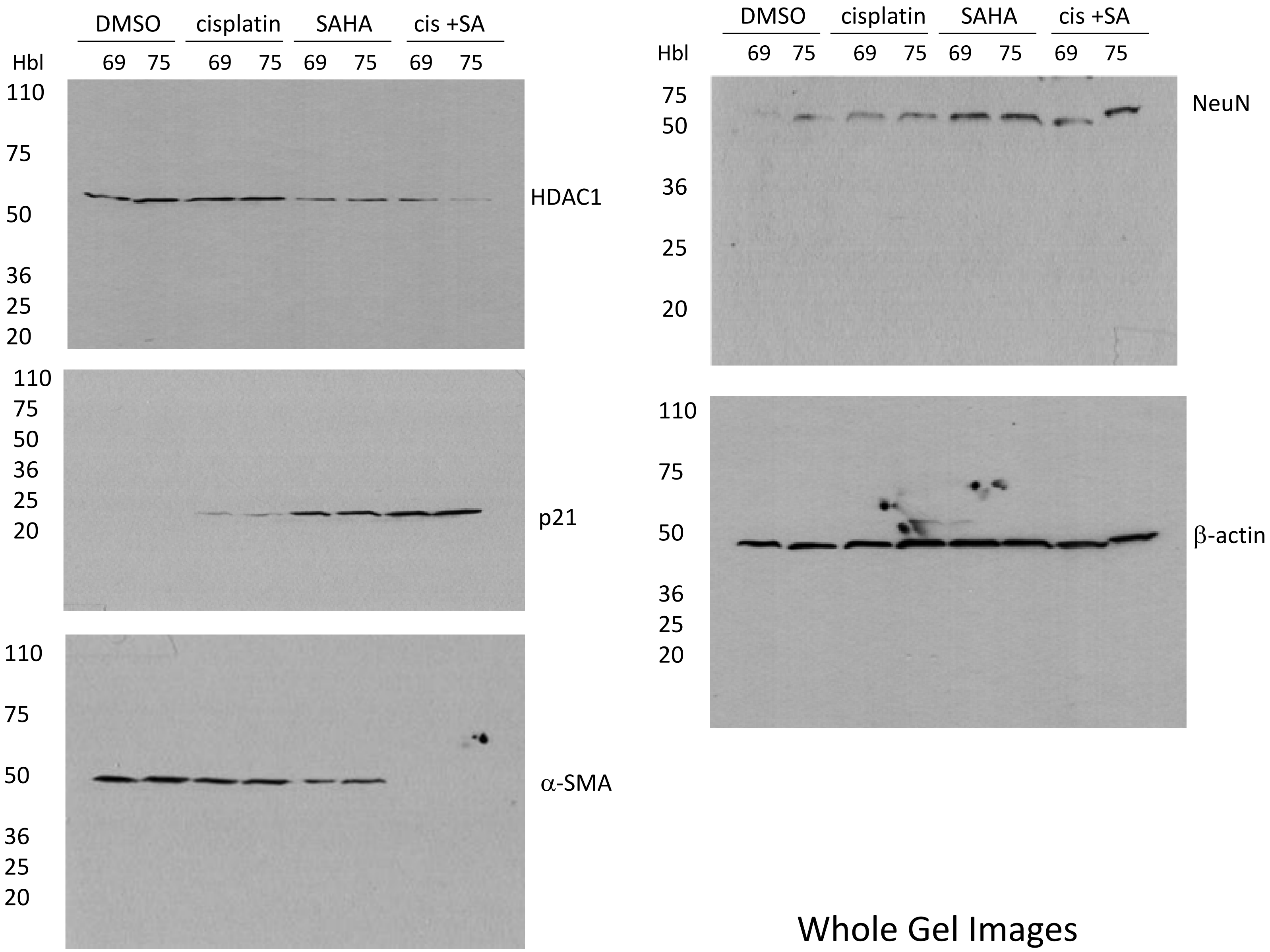

Whole Gel Images

Figure 5C

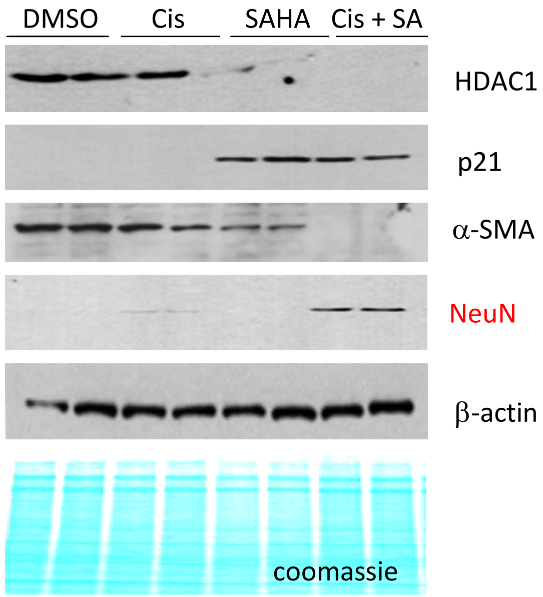

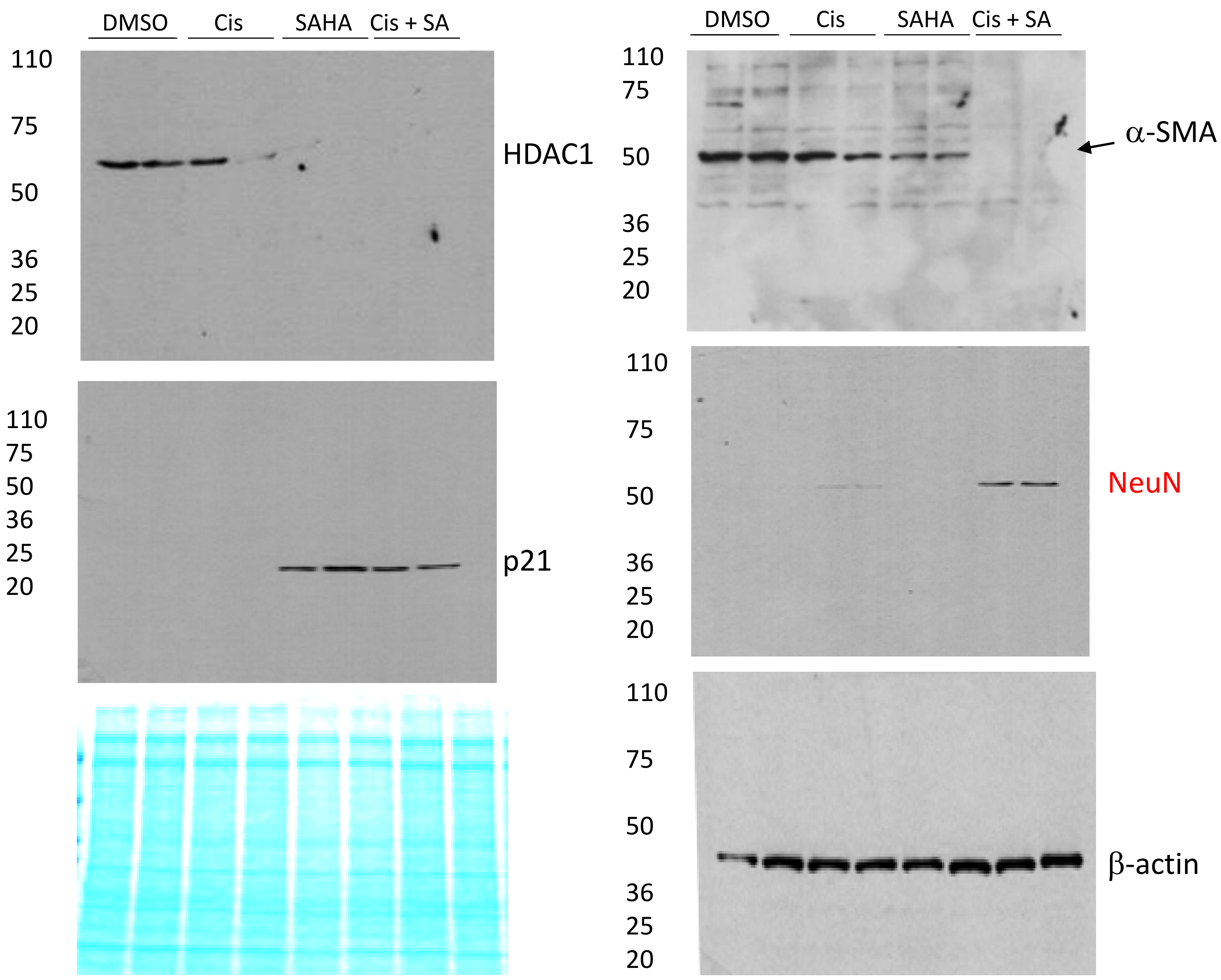

Whole Gel Images

Fig 5C bottom

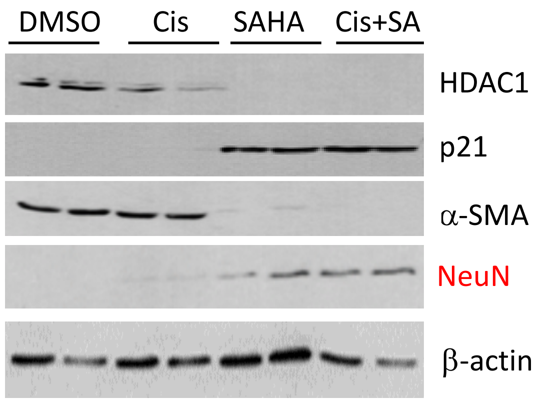

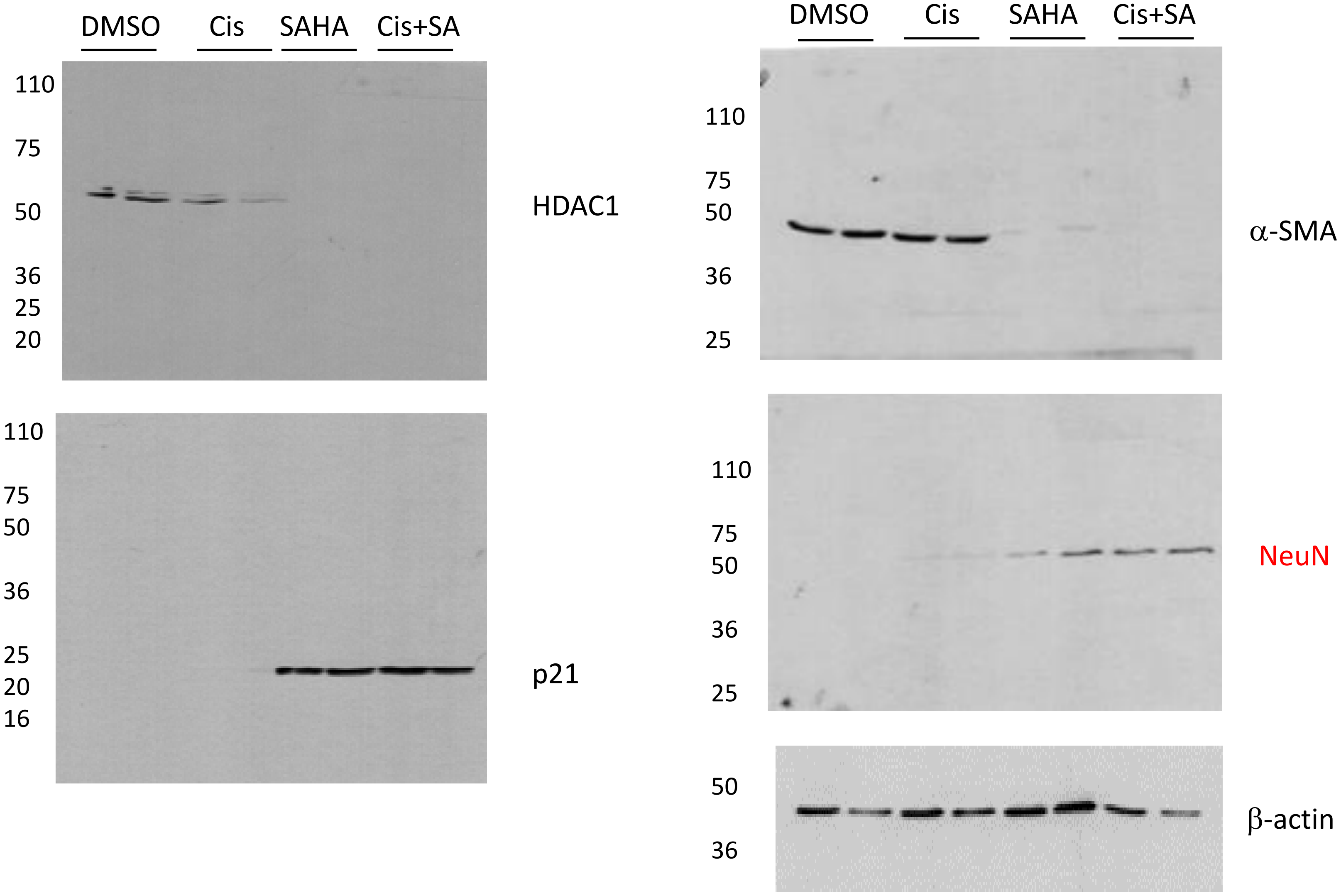

Whole Gel Images

Fig 5D

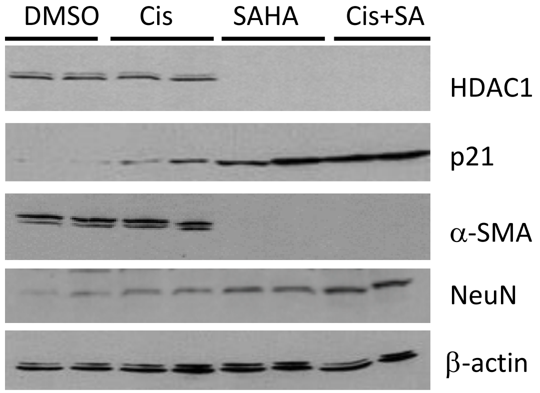

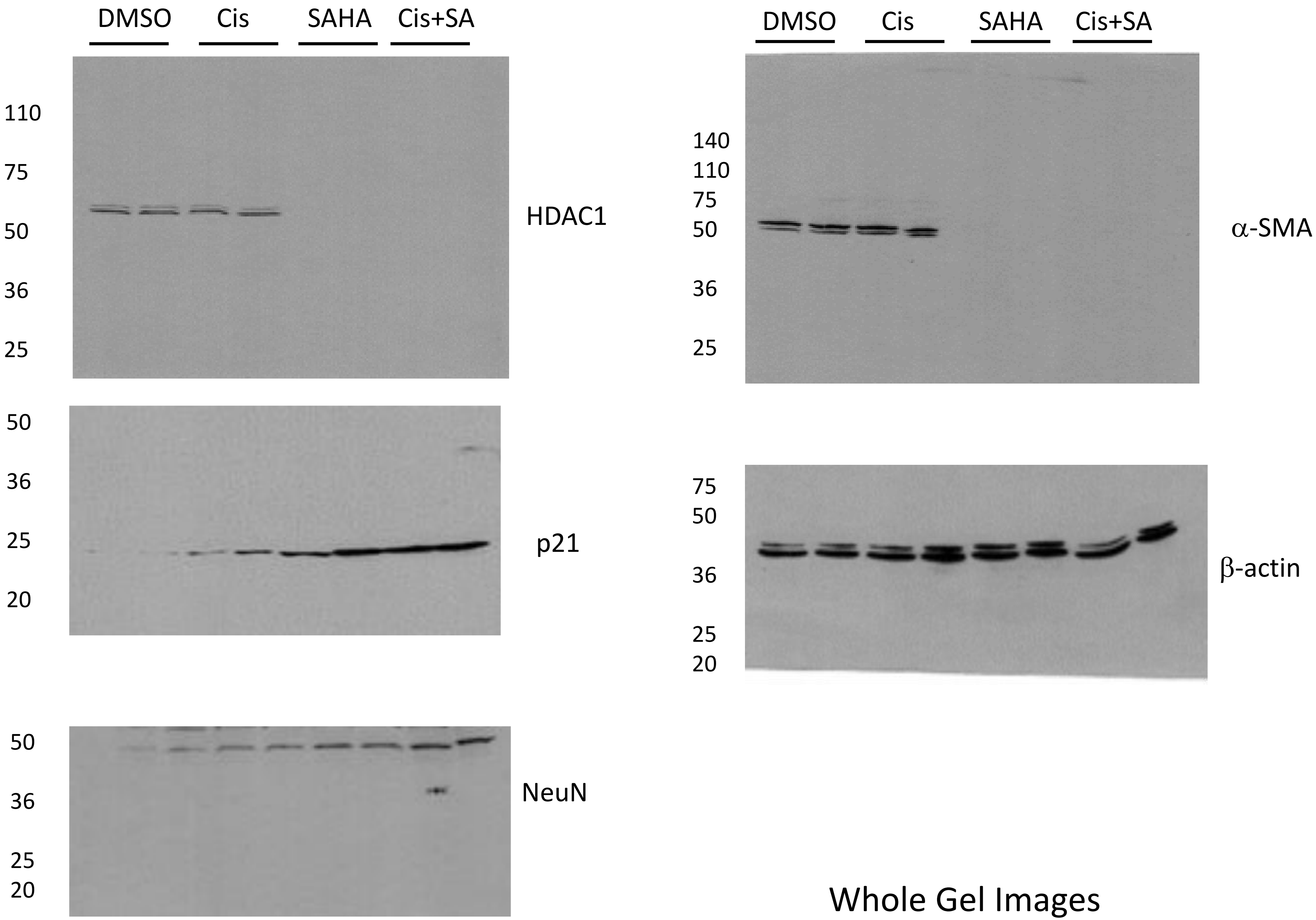

Whole Gel Images

Fig 6F

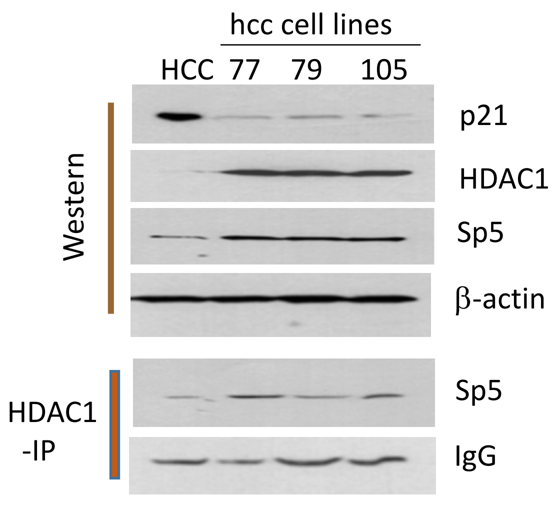

HDAC1-Sp5 complexes

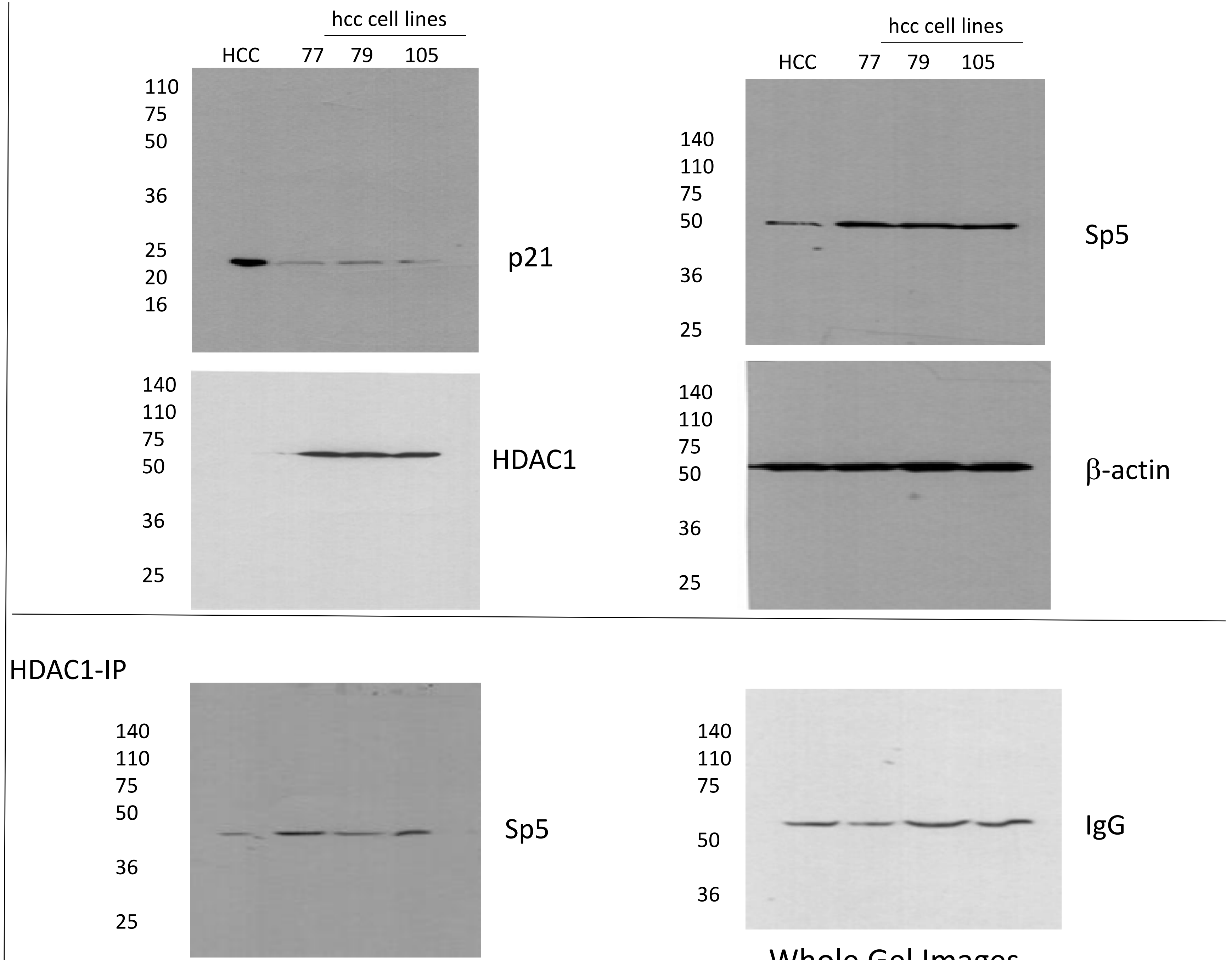

Whole Gel Images

Fig 7C

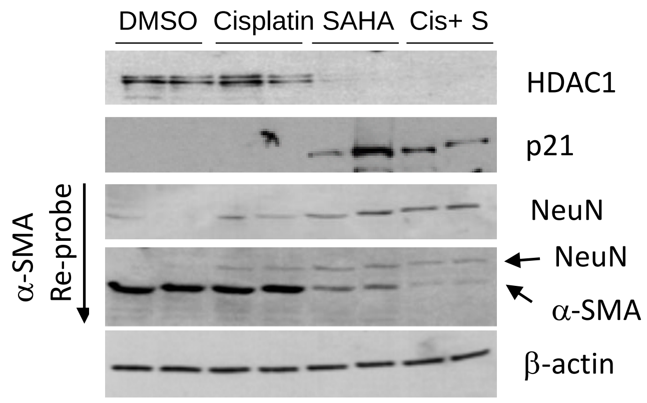

Upper

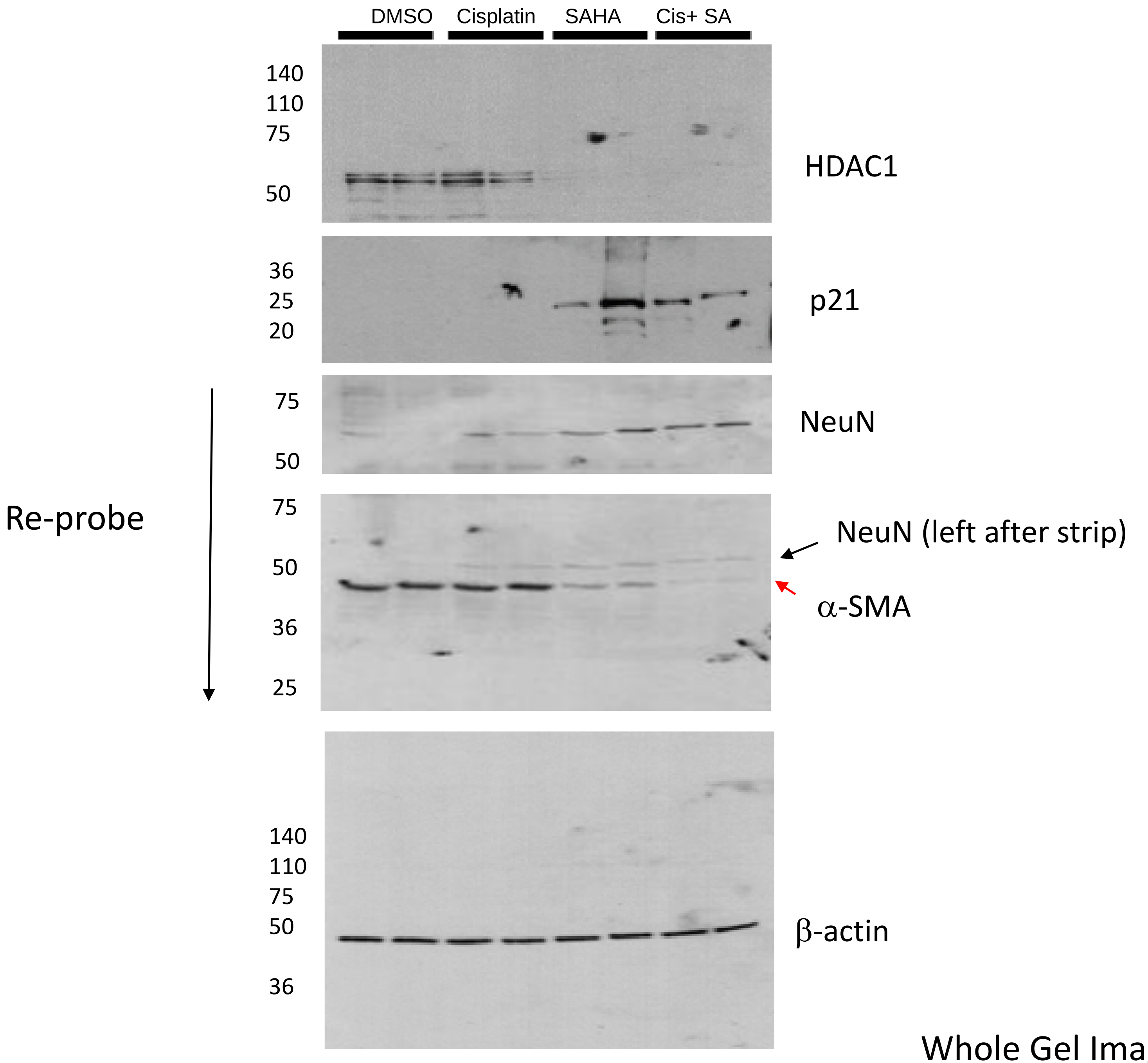

Whole Gel Images

Fig 7C

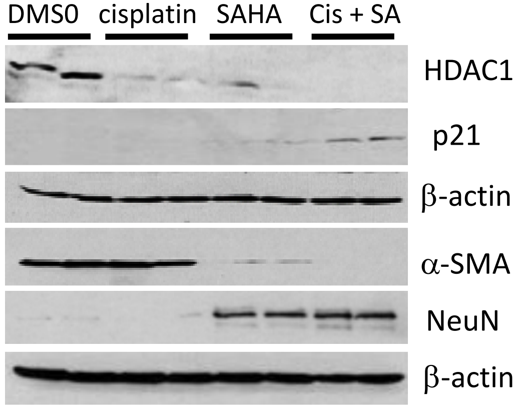

Middle

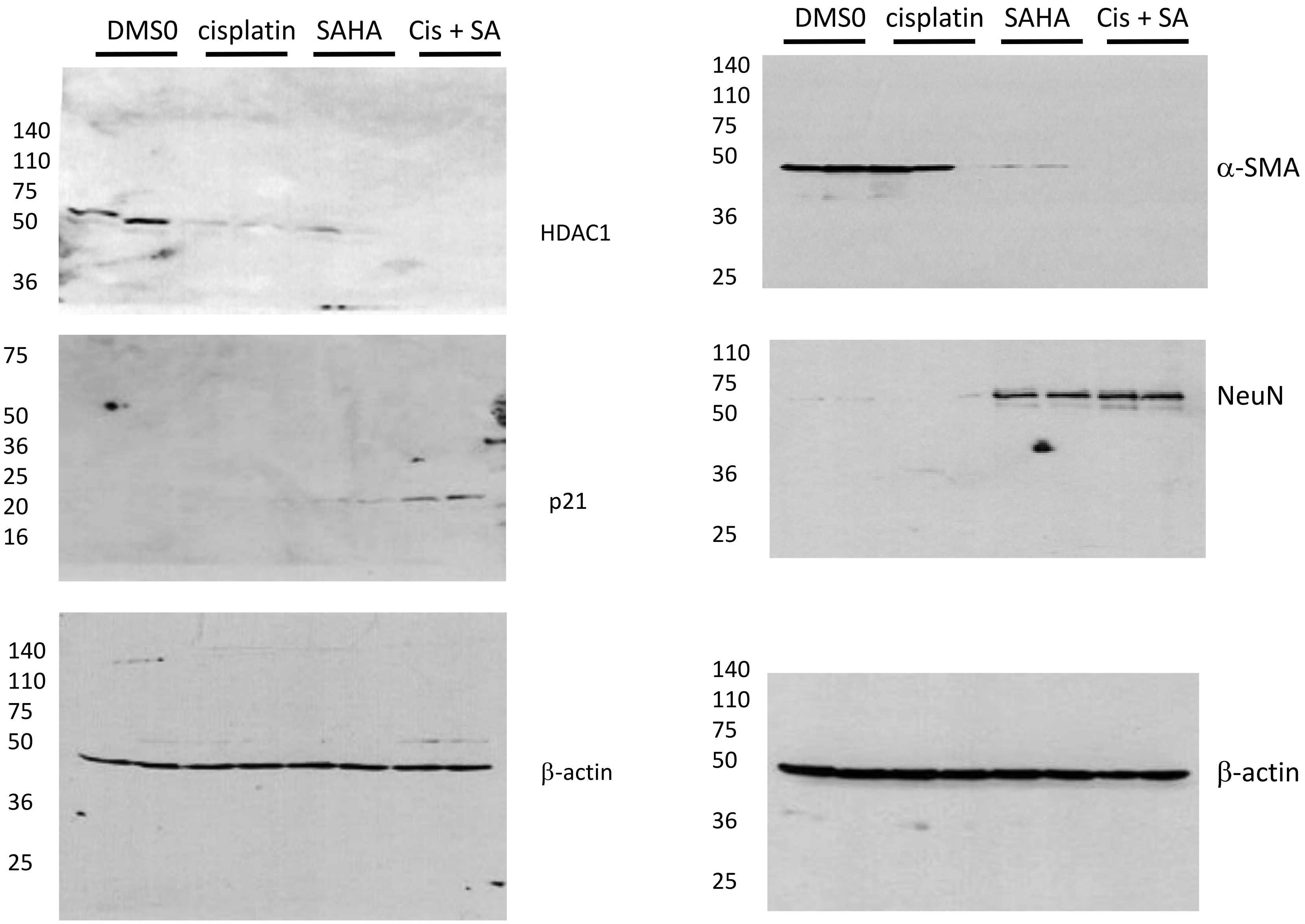

Whole Gel Images

Fig 7C

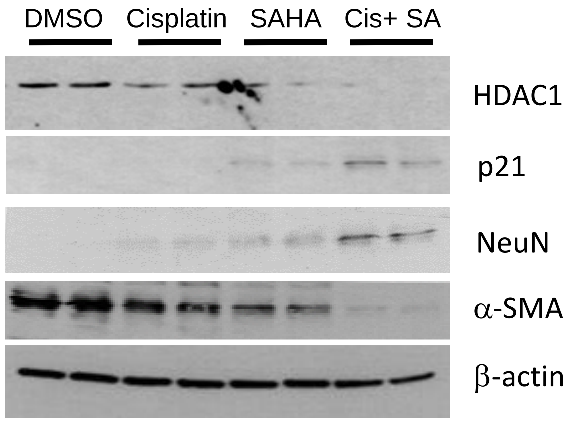

Bottom

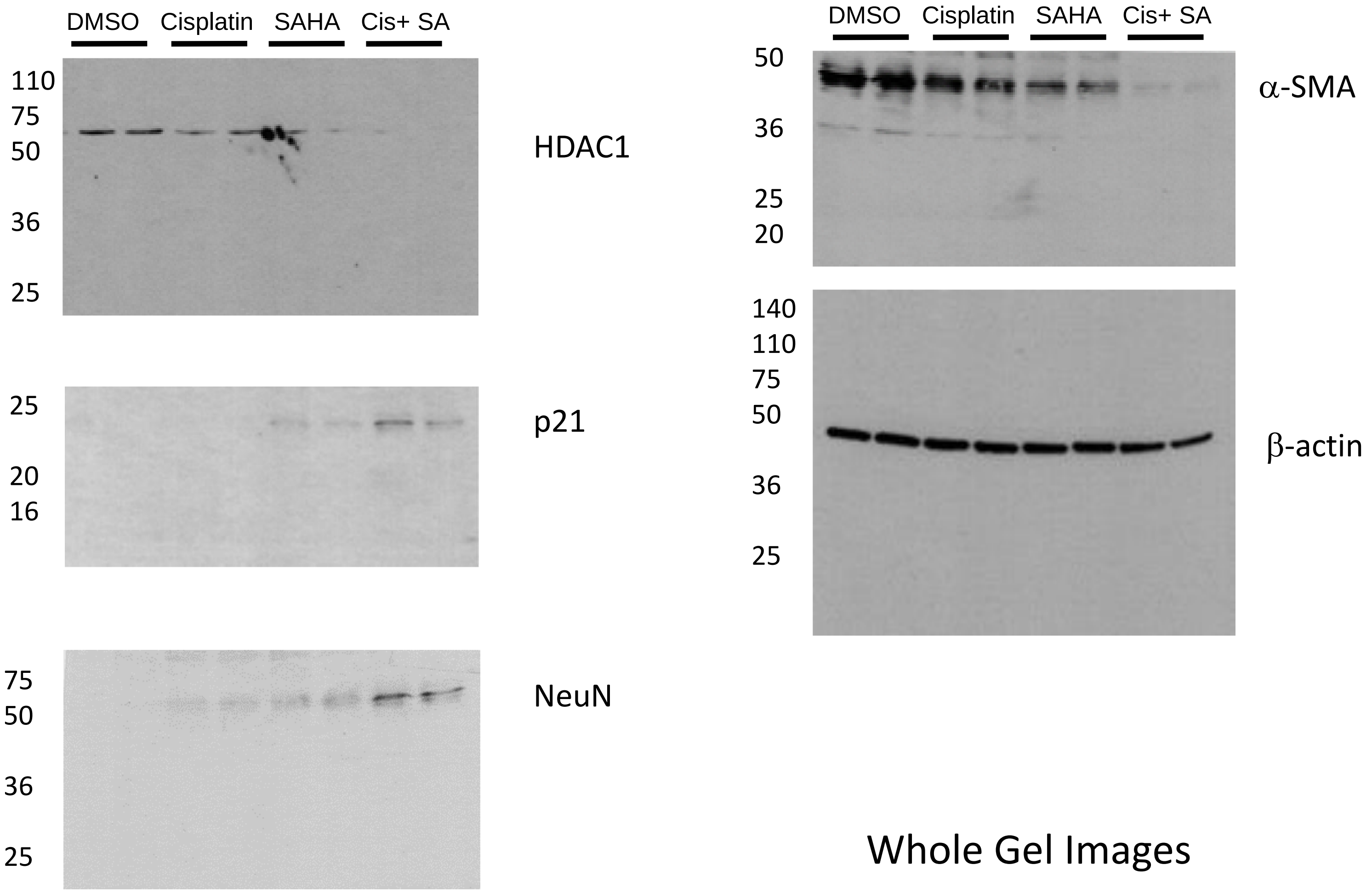

Whole Gel Images

Figure 8B

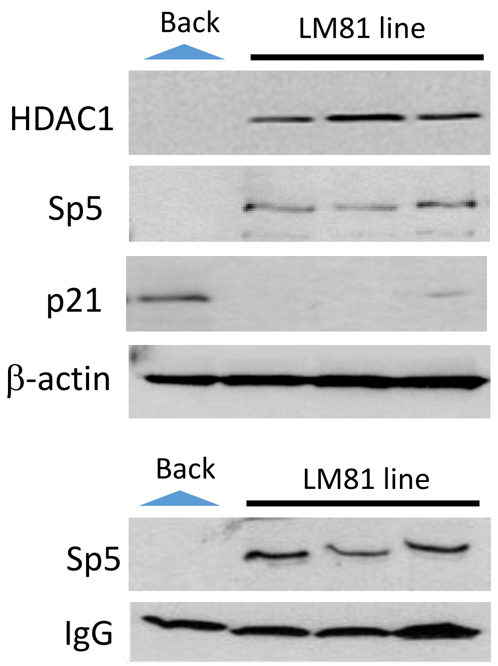

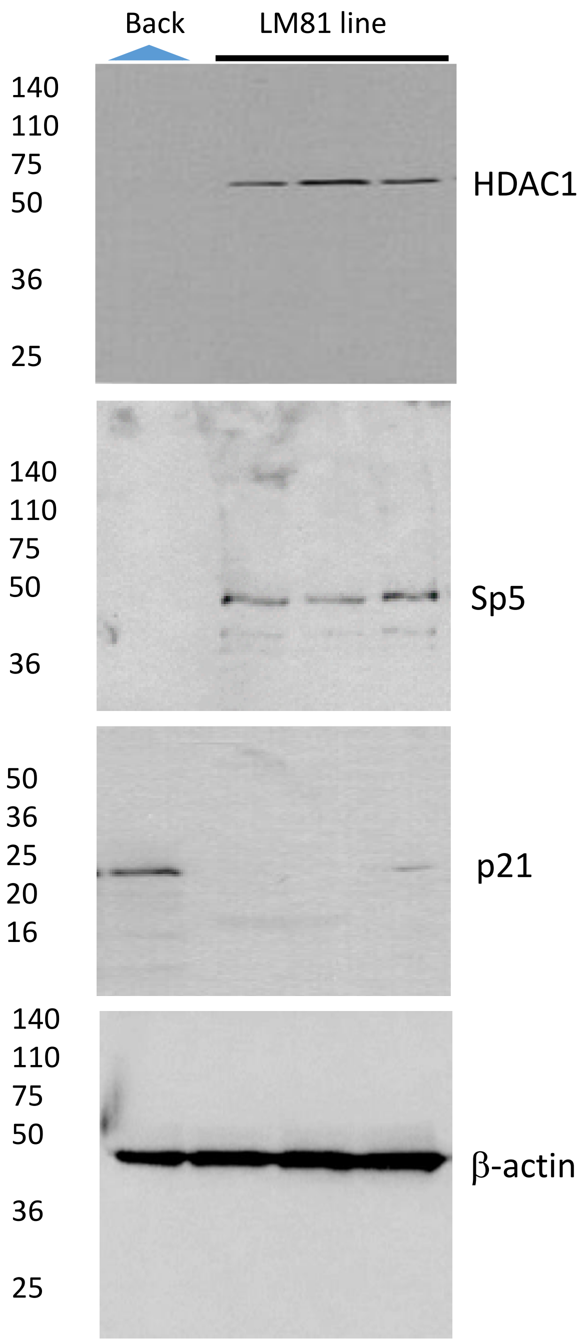

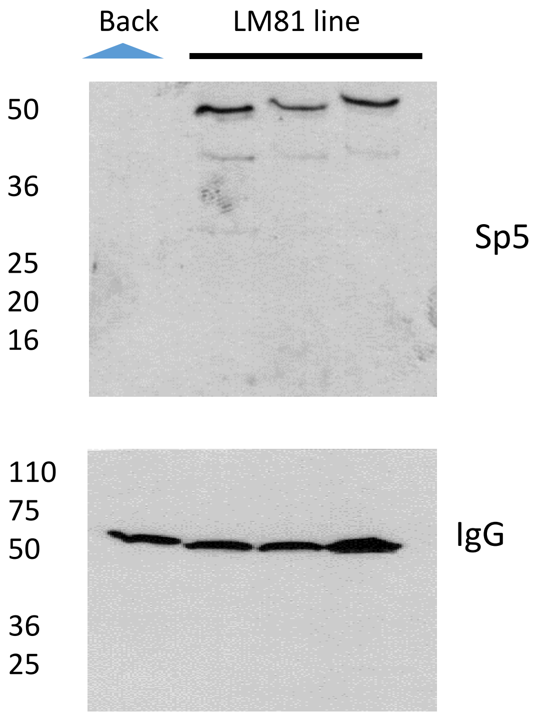

Whole Gel Images

Fig 8E

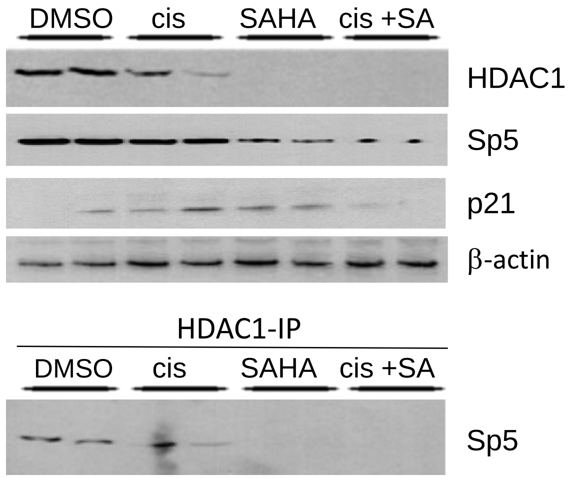

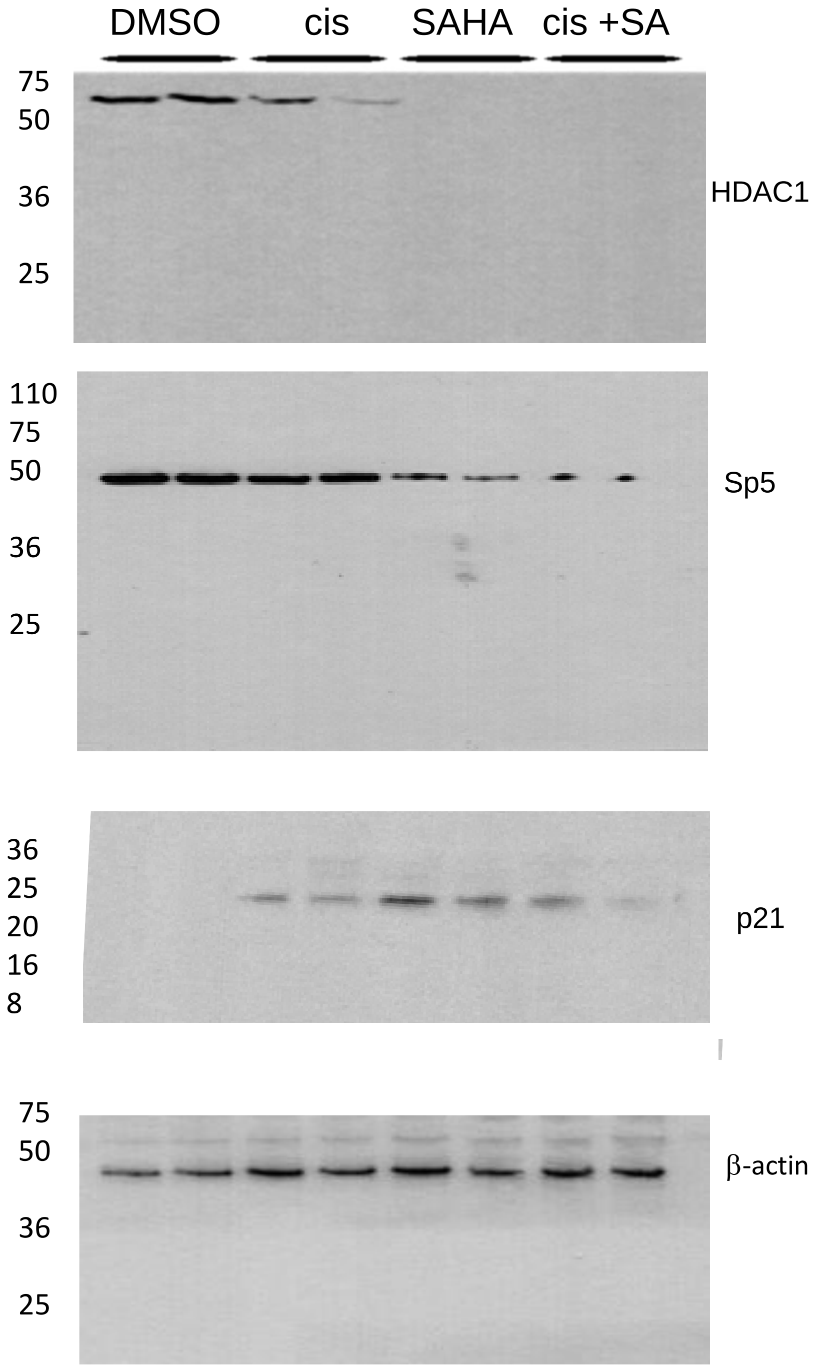

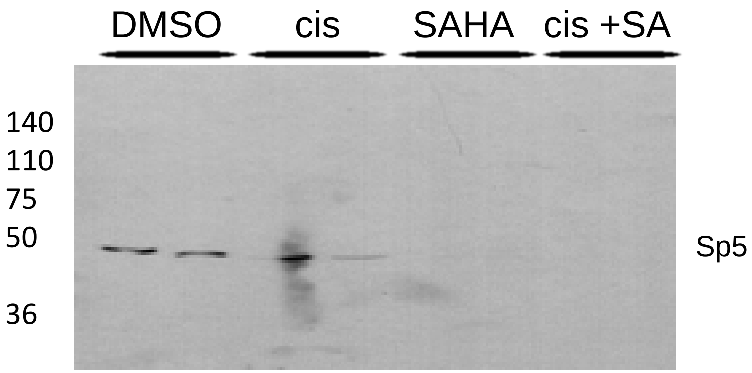

HDAC1-IP

Whole Gel Images

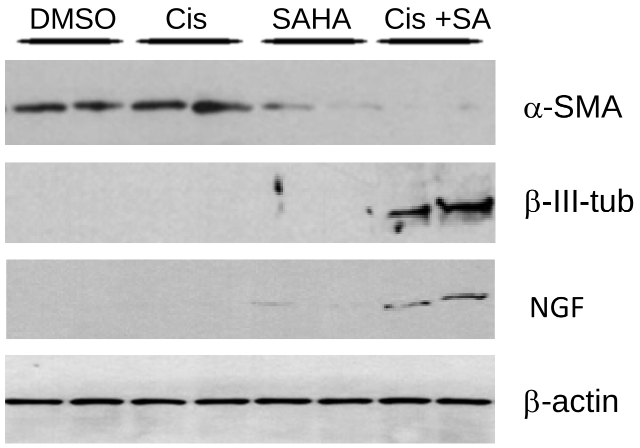

Fig 8F

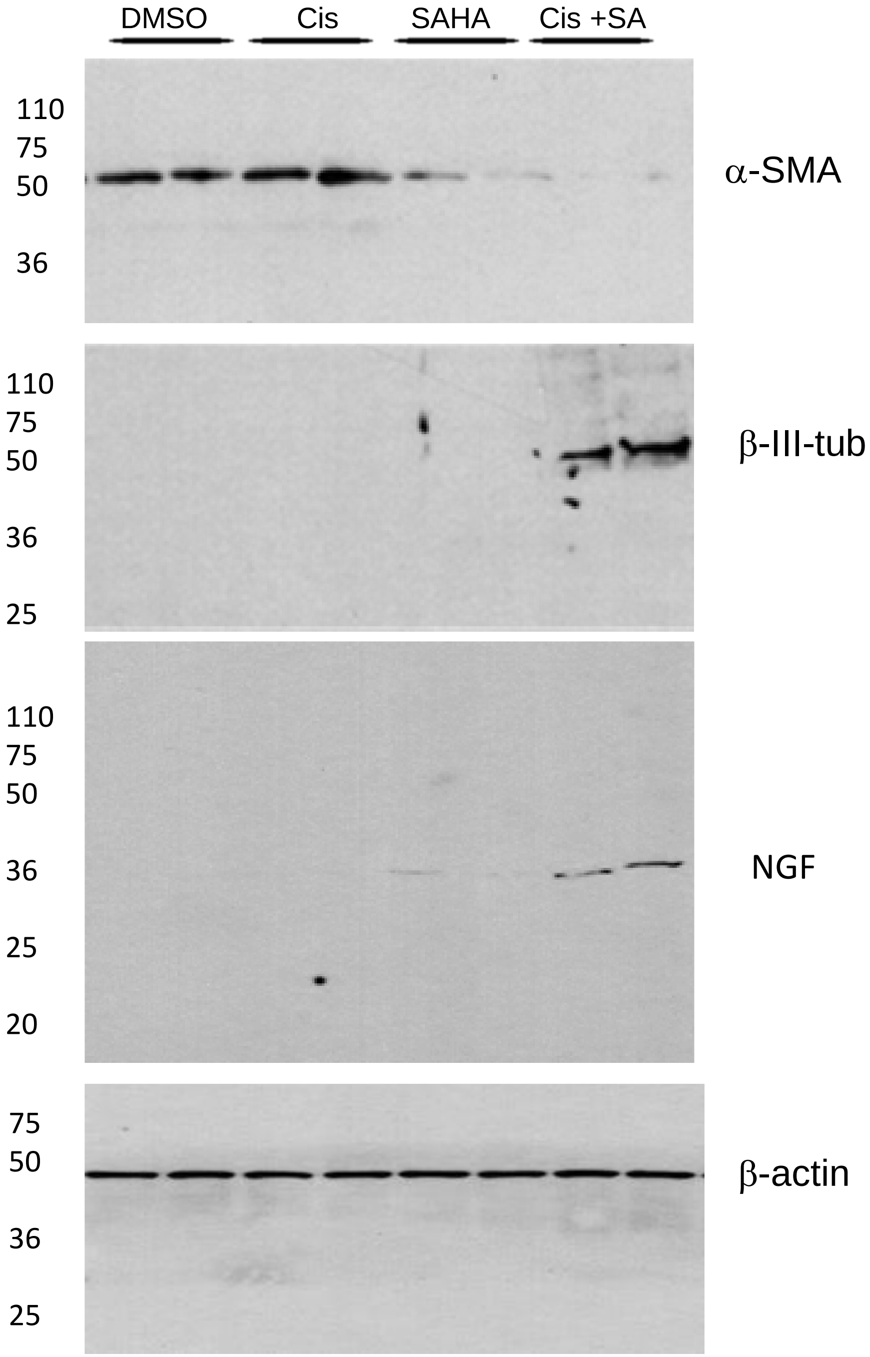

Whole Gel Images

Fig 9C

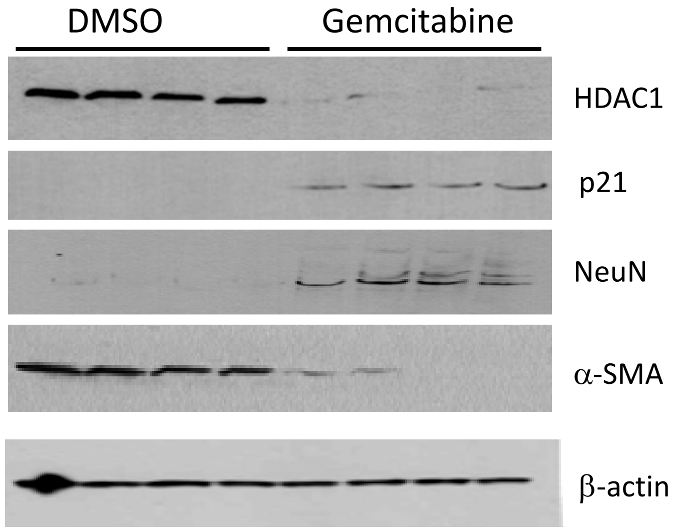

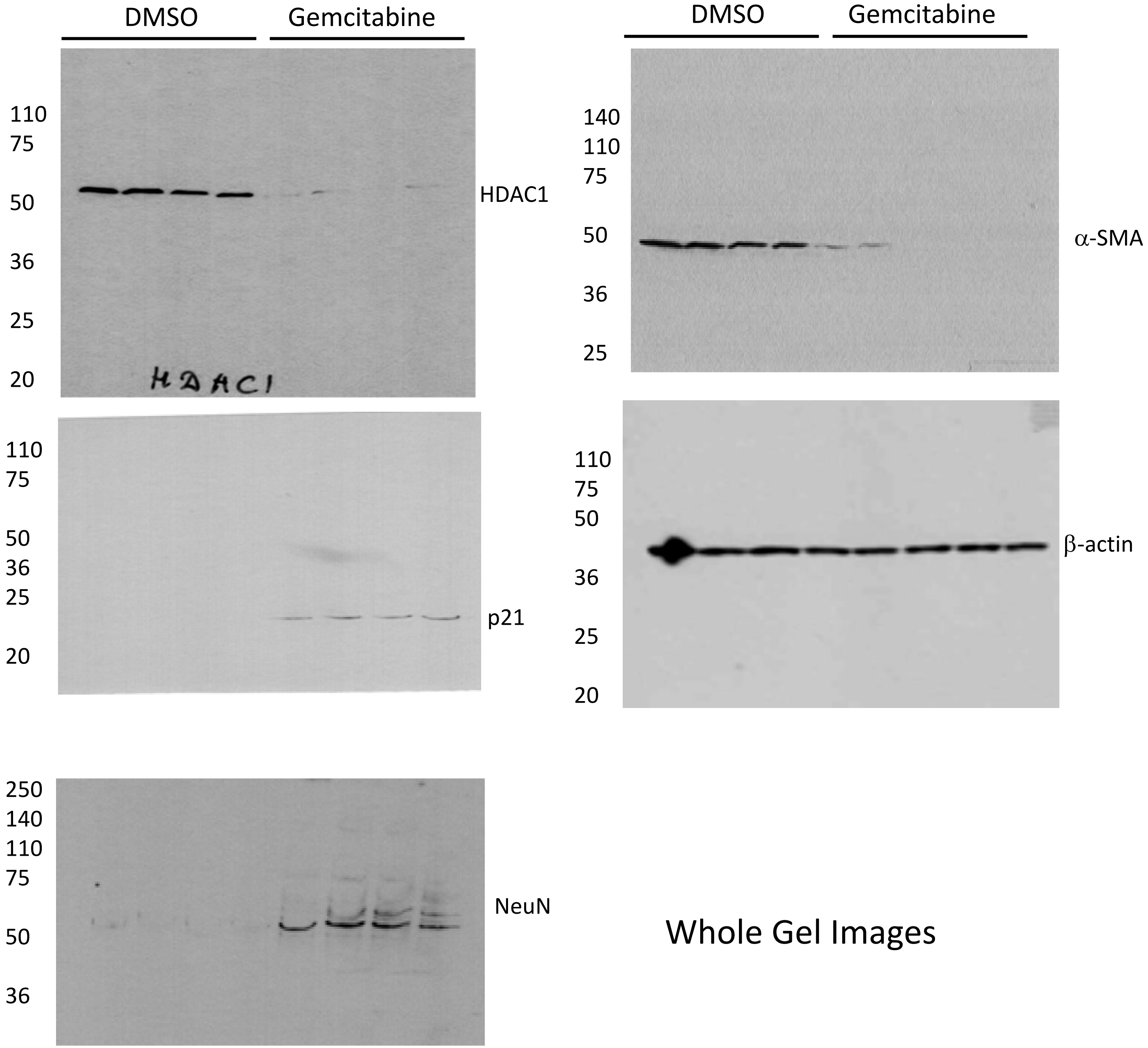

Whole Gel Images

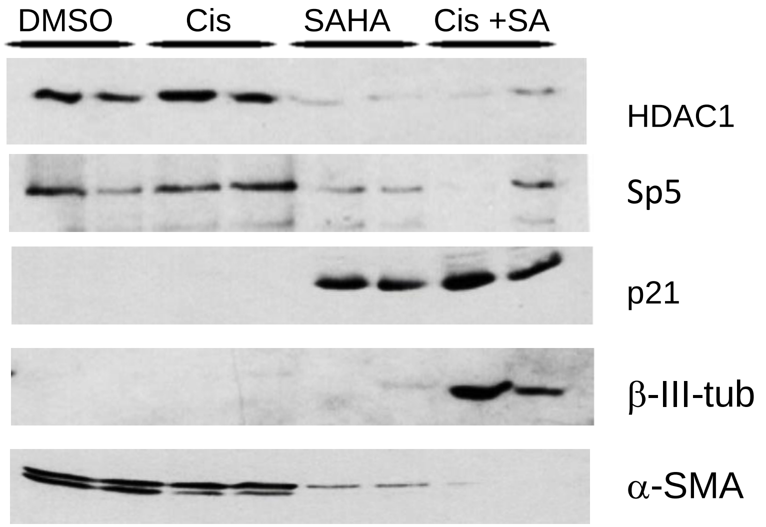

Supplemental Figure S1

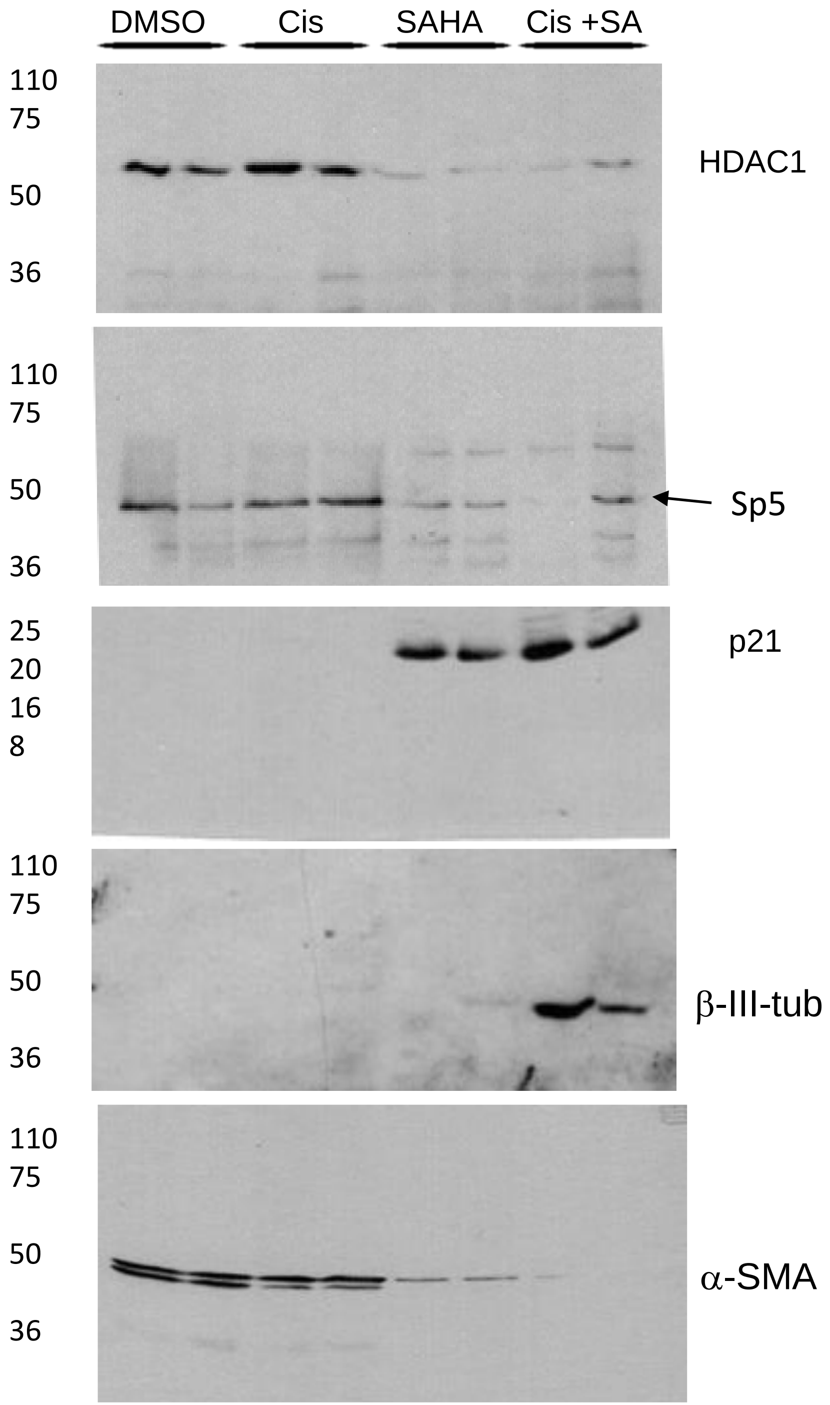

Whole Gel Images
